# Positive correlation between variants of lipid metabolism-related genes and coronary heart disease

**DOI:** 10.3892/mmr.2013.1454

**Published:** 2013-05-02

**Authors:** LI-NA ZHANG, PAN-PAN LIU, JIANQING ZHOU, R. STEPHANIE HUANG, FANG YUAN, LI-JUAN FEI, YI HUANG, LIMIN XU, LING-MEI HAO, XU-JUN QIU, YANPING LE, XI YANG, WEIFENG XU, XIAOYAN HUANG, MENG YE, JIANGFANG LIAN, SHIWEI DUAN

**Affiliations:** 1 School of Medicine, Ningbo University, Ningbo, Zhejiang 315211, P.R. China; 2 Ningbo Medical Center, Lihuili Hospital, Ningbo University, Ningbo, Zhejiang 315041, P.R. China; 3 Department of Medicine, University of Chicago, Chicago, IL 60637, USA; 4 Clinical Laboratory, The Seventh Hospital of Ningbo, Ningbo, Zhejiang 315202, P.R. China; 5 Department of Cardiology, The Affiliated Hospital, Ningbo University, Ningbo, Zhejiang 315211, P.R. China

**Keywords:** coronary heart disease, single nucleotide polymorphism, *APOB*, *LPA*, *LIPA*

## Abstract

Four gene variants related to lipid metabolism (including the rs562338 and rs503662 variants of the *APOB* gene, the rs7767084 variant of the *LPA* gene and the rs2246942 variant of the *LIPA* gene) have been shown to be associated with coronary heart disease (CHD). The aim of the present study was to assess their association with CHD in the Han Chinese population and to assess the contribution of these gene variants to CHD. Using the standardized coronary angiography method, we enrolled 290 CHD patients and 193 non-CHD patients as non-CHD controls from Lihuili Hospital (Ningbo, China). In addition, we recruited 330 unrelated healthy volunteers as healthy controls from the Xi Men Community (Ningbo, China). Our results demonstrated that the rs503662 and rs562338 variants of the *APOB* gene were extremely rare in the Han Chinese population (minor allele frequency <1%). Genotype rs2246942-GG of the *LIPA* gene was associated with an increased risk of CHD [CHD cases versus healthy controls: P=0.04; odds ratio (OR)=1.63; 95% confidence interval (CI)=1.02–2.60). Genotype rs7767084-CC of the *LPA* gene was identified as a protective factor against CHD in females (CHD cases versus non-CHD controls: P=0.04, OR=0.21; CHD cases versus healthy controls: P=0.02, OR=0.21). The results of our meta-analysis indicated that rs7767084 was not associated with a high risk of CHD (P=0.83; combined OR=0.93; 95% CI=0.47–1.85). In the present study, two single nucleotide polymorphisms (SNPs) of genes involved in lipid metabolism (rs2246942 and rs7767084) were identified to be significantly associated with CHD in the Han Chinese population. Specifically, rs2246942-GG of the *LIPA* gene was a risk factor for CHD, while rs7767084-CC of the *LPA* gene was a protective factor against CHD in females. However, our meta-analysis indicated that rs7767084 is not associated with a higher risk of CHD.

## Introduction

Cardiovascular disease (CVD) is the leading cause of human mortality worldwide. The prevalence and incidence of coronary heart disease (CHD) is increasing in numerous countries, including China (1). CHD is a complex disease that involves a variety of genetic and environmental factors. Increased concentrations of low-density lipoprotein cholesterol (LDL-C) in the blood is a well-established risk factor for CHD (2) and the primary target for lipid-lowering therapy in the prevention and treatment of CVD (3).

Apolipoprotein B (apoB) is the main apolipoprotein component of LDL-C and is important in the transport and metabolism of LDL-C. *APOB* gene variants (including rs562338 and rs503662) have been shown to be associated with an increased concentration of LDL-C in European and American populations (2,4,5). High levels of plasma apoB and LDL-C were also shown to increase the risk of CVD (6). A number of studies recommend the use of apoB instead of LDL-C as a predictor of CVD (7–9).

Cleaved fragments of lipoprotein(a) (LPA) protein are capable of attaching to atherosclerotic lesions and thus promote thrombogenesis (10,11). Elevated levels of plasma LPA are associated with atherosclerosis (12). *LPA* gene variants may contribute to the risk of CHD by regulating the level of lipids (13). SNP rs7767084 of the *LPA* gene was observed to be associated with levels of LDL-C (14) and CHD (15). However, rs7767084 of the *LPA* gene was not associated with CHD risk in the Hispanic population (16).

Lysosomal lipase A (LIPA) is able to catalyze the hydrolysis of cholesteryl esters and triglycerides. SNP rs2246942 of the *LIPA* gene was demonstrated to be significantly associated with the risk of CHD in European and South Asian populations (17). A recent study observed a significant association between rs1412444 of the *LIPA* gene and risk of CHD in South Asian and European populations (18).

The present study examined four gene variants involved in lipid metabolism: rs562338 and rs503662 of the *APOB* gene; rs7767084 of the *LPA* gene; and rs2246942 of the *LIPA* gene. We performed a case-control study to investigate their contribution to the risk of CHD in the Han Chinese population. A meta-analysis of three case-control studies among Han Chinese individuals was also performed to establish the role of *LPA* rs7767084 in CHD.

## Materials and methods

### Sample collection

A total of 483 unrelated inpatients were enrolled from Lihuili Hospital (Ningbo, Zhejiang, China). In addition, 330 healthy individuals (including 86 males and 244 females; mean age, 63.44±9.21 years) who originated from Ningbo City (China) were recruited as healthy controls. Patients were diagnosed by standardized coronary angiography according to Seldinger’s method (19), and assessed by at least two independent cardiologists. Patients with CHD (n=290; 209 males and 81 females; mean age, 61.98±9.49 years) demonstrated at least one of the following criteria: i) ≥50% coronary artery occlusion of one or more major coronary arteries (20); ii) a history of prior angioplasty; or iii) a history of coronary artery bypass surgery. Non-CHD controls (n=193; 98 males and 95 females; mean age, 58.65±9.36 years) with a <50% occlusion in the major coronary artery and no atherosclerotic vascular disease were selected from the inpatient population. All samples were obtained from individuals of Han Chinese ethnicity originating from Ningbo, China. Subjects with congenital heart disease, cardiomyopathy and liver or renal diseases were excluded from the study. Blood samples were collected in 3.2% citrate sodium-treated tubes and then stored at −80°C. The protocol of our study was approved by the ethical committee of Lihuili Hospital (Ningbo, Zhejiang, China). Written informed consent was obtained from all patients.

### SNP genotyping

Human genomic DNA was prepared from peripheral blood samples using the nucleic acid extraction automatic analyzer (Lab-Aid 820, Xiamen City, China) and was quantified using the PicoGreen^®^ double strand (dsDNA) DNA Quantification kit (Molecular Probes Inc., Eugene, USA). Amplification was performed on the ABI Geneamp^®^ PCR System 9700, Dual 384-Well Sample Block Module (Applied Biosystems, Foster City, CA) for the polymerase chain reaction (PCR). PCR conditions included an initial denaturation stage at 94°C for 15 sec, followed by 45 amplification cycles (including 94°C for 20 sec, 56°C for 30 sec and primer extension at 72°C for 1 min) and a final extension stage for 3 min at 72°C. Primer extension for genotyping was performed on the Sequenom^®^ Mass-ARRAY iPLEX^®^ (Sequenom, San Diego, CA, USA) platform according to the manufacturer’s instructions (21). The primer sequences of the four SNPs used for the PCR assays are shown in Table I.

### Meta-analysis

We systematically searched databases, including PubMed (http://www.ncbi.nlm.nih.gov/pubmed/) and the China National Knowledge Infrastructure (CNKI; http://www.cnki.net/) for all available case-control studies relating to rs7767084 of the *LPA* gene and CHD. We selected studies based on the following criteria: i) The study was an original study with an abstract in citation; ii) the study used a case-control or a prospective design; iii) the study contained complete data with genotype and allele frequencies; and iv) the genotype frequencies of controls were reported in Hardy-Weinberg equilibrium (HWE). Statistical heterogeneity between studies was estimated using the Q-test. An I^2^ value >50% indicated a significant heterogeneity among the studies included in the meta-analysis (22). A random-effects model based on the inverse-variance method was used for the studies with high heterogeneity. Publication bias was estimated using funnel plots (23).

### Statistical analysis

HWE was analyzed using the Arlequin program (version 3.5) (24). Genotype and allele frequencies were compared between CHD cases and each of the two controls using the PASW Statistics 18.0 software (SPSS, Inc., Somers, NY, USA). The odds ratio (OR) with 95% confidence interval (95% CI) were calculated using an online tool (http://faculty.vassar.edu/lowry/odds2x2.html). Power and Sample Size Calculation software (v3.0.43) was used to determine the power of the study (25). Correlation between genotype and the extent of CHD disease was also performed using the PASW Statistics 18.0 software. Meta-analyses were performed using RevMan software (version 5.1, Copenhagen: The Nordic Cochrane Centre, The Cochrane Collaboration, 2011). P<0.05 was considered to indicate a statistically significant result.

## Results

### Genetic tests

No departure from HWE was observed for the four lipid metabolism gene variants. SNPs rs503662 and rs562338 of the *APOB* gene were extremely rare in our samples (minor allele frequencies <1%), therefore they were not included in the further analysis. Genotype and allele frequencies of rs7767084 and rs2246942 are shown in Table II. No significant association with CHD was observed for the two SNPs. Further genetic tests under the recessive and dominant inheritance models were performed for rs7767084 and rs2246942, and the results of these tests are shown in Table III and Table IV, respectively. In the recessive model, a significant association between the rs2246942-GG genotype and risk of CHD was detected (CHD cases versus healthy controls: P=0.04; OR=1.63; 95% CI=1.02–2.60).

### CHD

CHD is the leading cause of human mortality worldwide. However, higher rates of CHD are observed in males compared with females across all age groups. In addition, coronary disease occurs up to 10 years later in females (26). Due to the genetic and habitual differences between genders, we performed a breakdown association test by gender to examine whether gender as a factor may influence the contribution of SNPs to CHD risk. Subsequently, we identified a significant association at the genotype level (Table V). Further tests under the dominant (Table IV) and recessive (Table III) models were also performed. In the recessive model, we observed a significant protective effect of rs7767084-CC against CHD in females (CHD cases versus non-CHD controls: P=0.04, OR=0.21, 95% CI=0.05–1.01; CHD cases versus healthy controls: P=0.02, OR=0.21, 95% CI=0.05–0.91). In addition, there was a correlation towards a significant association of rs2246942-GG with CHD in males under the recessive model (CHD cases versus healthy controls: P=0.06, OR=2.27, 95% CI=0.97–5.33). No significant association was identified in the dominant model.

**Figure 1:**
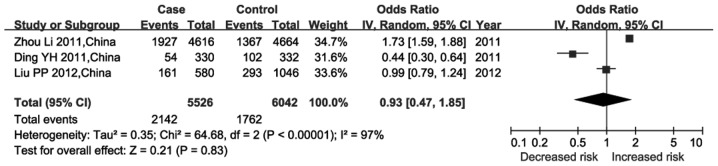
Meta-analysis of association studies between rs7767084 of the *LPA* gene and risk of CHD. CI, confidence interval; CHD, coronary heart disease.

**Figure 2:**
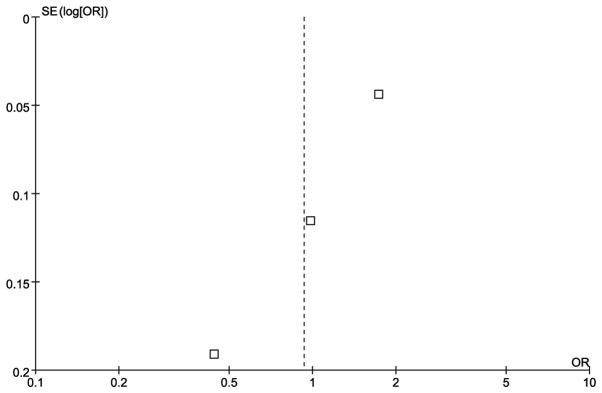
Funnel plots for studies in the meta-analysis.

In the CHD group, a correlation test was performed between the two gene variants and the number of coronary arteries with occlusion. No correlation between either of the two gene variants and CHD severity was observed (Table VI). For rs7767084 of the *LPA* gene, our meta-analysis included three case-control studies among the Han Chinese population (Fig. 1). The random-effects model was used since significant heterogeneity was observed among these studies (P<10^−5^; I^2^=97%). The results of the meta-analysis indicated that rs7767084 was not associated with risk of CHD (P=0.83; df=2; Z=0.21; combined OR=0.93; 95% CI=0.47–1.85). There was no publication bias according to the funnel plot (Fig. 2).

## Discussion

Two SNPs of the *APOB* gene (rs562338 and rs503662) were detected at extremely low levels in our samples. According to the information in the online HapMap dataset, the minor allele frequencies in the HapMap-HCB (Han Chinese in Beijing) are 1.1% for rs562338 and 1.1% for rs503662, in contrast with 22.5% and 31.7% in the HapMap-CEU (CEPH; Utah residents with ancestry from northern and western Europe), respectively (http://hapmap.ncbi.nlm.nih.gov/). These findings support our data and implicate a significant ethnic difference for the two SNPs. We were unable to observe a significant association between rs7767084 and the risk of CHD in the case-control study and the subsequent meta-analysis. This negative result in the Chinese population agrees with the previous results of a large-scale case-control study in the Hispanic population (16). Notably, a further breakdown test by gender demonstrated that the rs7767084-CC genotype acts as a protective factor against CHD in females under the recessive model (Table II). This gender-dependent result is novel and a further study on a larger scale is warranted. In the present study, genotype rs2246942-GG of the *LIPA* gene was shown to increase the risk of CHD by 63% in the recessive model (CHD cases versus healthy controls: P=0.04). In addition, a correlation between genotype rs2246942-GG with an increased risk of CHD was observed in males under the recessive model (CHD cases versus healthy controls: P=0.06, OR=2.27). Another SNP of the *LIPA* gene (rs2246833) is located only 968 bp away from rs2246942, and was previously implicated with an increased risk of CHD in European and South Asian populations (17). Our results suggest that rs2246942 of the *LIPA* gene is likely to contribute to the risk of CHD in the male Chinese population under the recessive model.

ApoB regulates the concentration of plasma LDL-C and is directly associated with CHD (27). Recent studies have shown that *APOB* polymorphisms (XbaI, MspI and 3′VNTR) are associated with the risk of CHD in the Chinese population (28,29). LPA may contribute to CVD through complex mechanisms that involve proatherogenic and prothrombotic pathways (30,31). LPA accumulates in the arterial wall of patients with CHD (32) and contributes to cholesterol deposition (33). Previous studies have reported that SNPs (rs10455872 and rs3798220) in the *LPA* region are associated with a higher risk of CHD (34–37). The *LIPA* gene encodes lysosomal acid lipase (LAL) (38,39), which hydrolyzes cholesteryl esters and triglycerides delivered to the lysosome. A loss of LAL function results in the accumulation of triglycerides and cholesteryl esters in the cell, and eventually causes the formation of atherosclerotic plaques (40). *LIPA* gene mutations may cause the cholesteryl ester storage disease and Wolman’s disease (41), which often accompany premature CVD.

CHD is a complex disease involving numerous genes. Although a total of 813 Han Chinese individuals were included in this study, it is not well powered for analyses, demonstrating that the power of the test under the recessive model is 53.5% for rs2246942-GG and 69% for rs7767084-CC in females. Meanwhile, the ratio of males and females enrolled in our sample requires adjustment to ensure a more balanced case-control study. All P-values provided in this study were not corrected by the number of tests, thus there is a chance that this study may include false positive results.

In conclusion, a gender-dependent association between rs7767084 of the *LPA* gene and CHD was observed in the female Chinese population under the recessive model. In addition, a possible explanation for the contribution of rs2246942-GG of the *LIPA* gene to the risk of CHD in the male Chinese population under the recessive model was identified.

## Figures and Tables

**Table I:** Primer sequences for the four SNPs.

SNP	Primer type	Primer sequence
rs562338	1st PCR primer	ACGTTGGATGCAGCCTAAATGTTCATTGTC
	2nd PCR primer	ACGTTGGATGCCATGGTTTGCATACATCAC
rs503662	1st PCR primer	ACGTTGGATGGATAGTATGTGTGGCAGAAG
	2nd PCR primer	ACGTTGGATGACCCTGAATCTAACACAATC
rs7767084	1st PCR primer	ACGTTGGATGTTGGGCTGGTCACTTTTGTC
	2nd PCR primer	ACGTTGGATGGTGACTCCAGAATGAAGCTC
rs2246942	1st PCR primer	ACGTTGGATGGGAAAGATCTCCAAGATAT
	2nd PCR primer	ACGTTGGATGCTTATTTTTCCCTTGCCTCC

SNP, single nucleotide polymorphism.

**Table II:** Distribution of genotype and allele frequencies between CHD cases and the two control groups.

A, SNP rs7767084											

	Genotype frequencies (%)						Allele frequencies (%)				
								
Group	TT	TC	CC	χ^2^	P-value	HWE	C	T	χ^2^	P-value	OR (95% CI)
CHD cases	149 (51.4)	121 (41.7)	20 (6.9)			0.49	161 (27.8)	419 (72.2)			
Control 1	105 (54.4)	74 (38.3)	14 (7.3)	0.55	0.76	0.85	102 (26.4)	284 (73.6)	0.21	0.65	1.07 (0.80, 1.43)
Control 2	171 (51.8)	127 (38.5)	32 (9.7)	1.85	0.40	0.24	191 (28.9)	469 (71.1)	0.21	0.65	0.94 (0.74, 1.21)

B, SNP rs2246942											

	Genotype frequencies (%)						Allele frequencies (%)				
								
Group	AA	AG	GG	χ^2^	P-value	HWE	G	A	χ^2^	P-value	OR (95% CI)

CHD cases	115 (39.7)	128 (44.1)	47 (16.2)			0.26	222 (38.3)	358 (61.7)			
Control 1	69 (35.8)	96 (49.7)	28 (14.5)	1.46	0.48	0.56	152 (39.4)	234 (60.6)	0.12	0.73	0.96 (0.73, 1.24)
Control 2	136 (41.3)	158 (48.0)	35 (10.7)	4.22	0.12	0.27	228 (34.7)	430 (65.3)	1.75	0.19	1.17 (0.93, 1.48)

Control 1, non-CHD controls; control 2, healthy controls; CHD, coronary heart disease; SNP, single nucleotide polymorphism; HWE, Hardy-Weinberg equilibrium; OR, odds ratio; CI, confidence interval.

**Table III:** Genetic analysis of the two gene variants under the recessive model.

A, SNP rs7767084				

	Genotype frequencies			
			
Group	CC	TT+TC	P-value	OR (95% CI)
Total				
CHD cases	20	270		
Control 1	14	179	0.88	0.95 (0.47, 1.92)
Control 2	32	298	0.21	0.69 (0.39, 1.24)
Male				
CHD cases	18	191		
Control 1	4	94	0.15	2.21 (0.73, 6.73)
Control 2	6	80	0.64	1.26 (0.48, 3.28)
Female				
CHD cases	2	79		
Control 1	10	85	0.04	0.21 (0.05, 1.01)
Control 2	26	218	0.02	0.21 (0.05, 0.91)

B, SNP rs2246942				

	Genotype frequencies			
			
Group	GG	AA+AG	P-value	OR (95% CI)

Total				
CHD cases	47	243		
Control 1	28	165	0.61	1.14 (0.69, 1.89)
Control 2	35	294	0.04	1.63 (1.02, 2.60)
Male				
CHD cases	35	174		
Control 1	14	84	0.58	1.21 (0.62, 2.36)
Control 2	7	79	0.06	2.27 (0.97, 5.33)
Female				
CHD cases	12	69		
Control 1	14	81	0.99	1.01 (0.44, 2.32)
Control 2	28	215	0.43	1.33 (0.64, 2.77)

Control 1, non-CHD controls; control 2, healthy controls; CHD, coronary heart disease; SNP, single nucleotide polymorphism; OR, odds ratio; CI, confidence interval.

**Table IV:** Genetic analysis of the two gene variants under the dominant model.

A, SNP rs7767084				

	Genotype frequencies			
			
Group	TC+CC	TT	P-value	OR (95% CI)
Total				
CHD cases	141	149		
Control 1	88	105	0.51	1.13 (0.78, 1.63)
Control 2	159	171	0.91	1.02 (0.74, 1.40)
Male				
CHD cases	98	111		
Control 1	46	52	0.99	1.00 (0.62, 1.61)
Control 2	38	48	0.67	1.11 (0.67, 1.85)
Female				
CHD cases	43	38		
Control 1	42	53	0.24	1.43 (0.79, 2.59)
Control 2	121	123	0.59	1.15 (0.69, 1.90)

B, SNP rs2246942				

	Genotype frequencies			
			
Group	AG+GG	AA	P-value	OR (95% CI)

Total				
CHD cases	175	115		
Control 1	124	69	0.39	0.85 (0.58, 1.23)
Control 2	193	136	0.67	1.07 (0.78, 1.48)
Male				
CHD cases	118	91		
Control 1	64	34	0.14	0.69 (0.42, 1.13)
Control 2	49	37	0.93	0.98 (0.59, 1.63)
Female				
CHD cases	57	24		
Control 1	60	35	0.31	1.38 (0.73, 2.61)
Control 2	144	99	0.07	1.63 (0.95, 2.81)

Control 1, non-CHD controls; control 2, healthy controls; CHD, coronary heart disease; SNP, single nucleotide polymorphism; OR, odds ratio; CI, confidence interval.

**Table V:** Genetic testing of the two gene variants stratified by gender.

A, SNP rs7767084											

	Genotype frequencies (%)						Allele frequencies (%)				
								
Group	TT	TC	CC	χ^2^	P-value	HWE	C	T	χ ^2^	P-value	OR (95% CI)
Male											
CHD cases	111	80	18			0.51	116	302			
Control 1	52	42	4	2.26	0.32	0.21	50	146	0.34	0.56	1.12 (0.76, 1.65)
Control 2	48	32	6	0.30	0.86	0.83	44	128	0.29	0.59	1.12 (0.75, 1.67)
Female											
CHD cases	38	41	2			0.02	45	117			
Control 1	53	32	10	7.85	0.02	0.14	52	138	0.01	0.93	1.02 (0.64, 1.63)
Control 2	123	95	26	6.86	0.03	0.24	147	341	0.32	0.57	0.89 (0.60, 1.32)

B, SNP rs2246942											

	Genotype frequencies (%)						Allele frequencies (%)				
								
Group	AA	AG	GG	χ ^2^	P-value	HWE	G	A	χ ^2^	P-value	OR (95% CI)

Male											
CHD cases	91	83	35			0.04	153	265			
Control 1	34	50	14	3.51	0.17	0.52	78	118	0.58	0.45	0.87 (0.62, 1.24)
Control 2	37	42	7	4.37	0.11	0.30	56	116	0.87	0.35	1.20 (0.82, 1.74)
Female											
CHD cases	24	45	12			0.22	69	93			
Control 1	35	46	14	1.11	0.57	0.86	74	116	0.48	0.49	1.16 (0.76, 1.78)
Control 2	99	116	28	3.26	0.20	0.49	172	314	2.70	0.10	1.35 (0.94, 1.95)

Control 1, non-CHD controls; control 2, healthy controls; CHD, coronary heart disease; SNP, single nucleotide polymorphism; OR, odds ratio; CI, confidence interval.

**Table VI:** Correlation between two gene variants and the number of stenoses in CHD cases under the dominant and recessive models.

		Number of stenoses			
			
Model	N	1	2	≥3	P-value
Dominant					
rs7767084					
TT	129	43	32	54	0.23
TC+CC	121	51	25	45	
rs2246942					
AA	98	34	24	40	0.56
AG+GG	152	60	33	59	
Recessive					
rs7767084					
CC	15	5	1	9	0.26
TC+TT	235	89	56	90	
rs2246942					
GG	41	18	13	10	0.08
AG+AA	209	76	44	89	

CHD, coronary heart disease.

## References

[1] Yu J, Huang J, Liang Y (2011). Lack of association between apolipoprotein C3 gene polymorphisms and risk of coronary heart disease in a Han population in East China. Lipids Health Dis.

[2] Sandhu MS, Waterworth DM, Debenham SL (2008). LDL-cholesterol concentrations: a genome-wide association study. Lancet.

[3] Baigent C, Keech A, Kearney PM (2005). Efficacy and safety of cholesterol-lowering treatment: prospective meta-analysis of data from 90,056 participants in 14 randomised trials of statins. Lancet.

[4] Willer CJ, Sanna S, Jackson AU (2008). Newly identified loci that influence lipid concentrations and risk of coronary artery disease. Nat Genet.

[5] Lettre G, Palmer CD, Young T (2011). Genome-wide association study of coronary heart disease and its risk factors in 8,090 African Americans: the NHLBI CARe Project. PLoS Genet.

[6] St-Pierre AC, Cantin B, Dagenais GR (2006). Apolipoprotein-B, low-density lipoprotein cholesterol, and the long-term risk of coronary heart disease in men. Am J Cardiol.

[7] Moss AJ, Goldstein RE, Marder VJ (1999). Thrombogenic factors and recurrent coronary events. Circulation.

[8] Talmud PJ, Hawe E, Miller GJ, Humphries SE (2002). Nonfasting apolipoprotein B and triglyceride levels as a useful predictor of coronary heart disease risk in middle-aged UK men. Arterioscler Thromb Vasc Biol.

[9] Walldius G, Jungner I (2006). The apoB/apoA-I ratio: a strong, new risk factor for cardiovascular disease and a target for lipid-lowering therapy - a review of the evidence. J Intern Med.

[10] Kamstrup PR, Tybjærg-Hansen A, Nordestgaard BG (2012). Genetic evidence that lipoprotein(a) associates with atherosclerotic stenosis rather than venous thrombosis. Arterioscler Thromb Vasc Biol.

[11] Szilágyi S, Péter A, Magyar MT (2012). Recurrent arterial thrombosis associated with the antithrombin basel variant and elevated lipoprotein(a) plasma level in an adolescent patient. J Pediatr Hematol Oncol.

[12] Terres W, Tatsis E, Pfalzer B (1995). Rapid angiographic progression of coronary artery disease in patients with elevated lipoprotein(a). Circulation.

[13] Deshmukh HA, Colhoun HM, Johnson T (2012). Genome-wide association study of genetic determinants of LDL-c response to atorvastatin therapy: importance of Lp(a). J Lipid Res.

[14] Teslovich TM, Musunuru K, Smith AV (2010). Biological, clinical and population relevance of 95 loci for blood lipids. Nature.

[15] Trégouët DA, König IR, Erdmann J (2009). Genome-wide haplotype association study identifies the SLC22A3-LPAL2-LPA gene cluster as a risk locus for coronary artery disease. Nat Genet.

[16] Qi L, Ma J, Qi Q (2011). Genetic risk score and risk of myocardial infarction in Hispanics. Circulation.

[17] Butterworth AS, Braund PS, Farrall M, IBC 50K CAD Consortium (2011). Large-scale gene-centric analysis identifies novel variants for coronary artery disease. PLoS Genet.

[18] Peden JF, Hopewell JC, Saleheen D (2011). Coronary Artery Disease (C4D) Genetics Consortium: A genome-wide association study in Europeans and South Asians identifies five new loci for coronary artery disease. Nat Genet.

[19] Higgs ZC, Macafee DA, Braithwaite BD, Maxwell-Armstrong CA (2005). The Seldinger technique: 50 years on. Lancet.

[20] Reilly MP, Li M, He J (2011). Identification of ADAMTS7 as a novel locus for coronary atherosclerosis and association of ABO with myocardial infarction in the presence of coronary atherosclerosis: two genome-wide association studies. Lancet.

[21] Gabriel S, Ziaugra L, Tabbaa D (2009). SNP genotyping using the Sequenom MassARRAY iPLEX platform. Curr Protoc Hum Genet.

[22] Higgins JP, Thompson SG, Deeks JJ, Altman DG (2003). Measuring inconsistency in meta-analyses. BMJ.

[23] Egger M, Davey Smith G, Schneider M, Minder C (1997). Bias in meta-analysis detected by a simple, graphical test. BMJ.

[24] Excoffier L, Lischer HE (2010). Arlequin suite ver 3.5: a new series of programs to perform population genetics analyses under Linux and Windows. Mol Ecol Resour.

[25] Dupont WD, Plummer WD (1990). Power and sample size calculations. A review and computer program. Control Clin Trials.

[26] Emslie C (2005). Women, men and coronary heart disease: a review of the qualitative literature. J Adv Nurs.

[27] Robinson JG, Wang S, Jacobson TA (2012). Meta-analysis of comparison of effectiveness of lowering apolipoprotein B versus low-density lipoprotein cholesterol and nonhigh-density lipoprotein cholesterol for cardiovascular risk reduction in randomized trials. Am J Cardiol.

[28] Li S, Lei ZW, Chen Z (2003). Relationship between apolipoprotein E and apolipoprotein B polymorphisms in youths with coronary heart disease. Zhonghua Yi Xue Yi Chuan Xue Za Zhi.

[29] Huang G, Zhong H, Re HM (2012). Coalition of DNA polymorphisms of ApoB and ApoAI genes is related with coronary artery disease in Kazaks. J Geriatr Cardiol.

[30] Boffa MB, Marcovina SM, Koschinsky ML (2004). Lipoprotein(a) as a risk factor for atherosclerosis and thrombosis: mechanistic insights from animal models. Clin Biochem.

[31] Kamstrup PR (2010). Lipoprotein(a) and ischemic heart disease - a causal association? A review. Atherosclerosis.

[32] Rath M, Niendorf A, Reblin T (1989). Detection and quantification of lipoprotein(a) in the arterial wall of 107 coronary bypass patients. Arteriosclerosis.

[33] Kiechl S, Willeit J (2010). The mysteries of lipoprotein(a) and cardiovascular disease revisited. J Am Coll Cardiol.

[34] Clarke R, Peden JF, Hopewell JC, PROCARDIS Consortium (2009). Genetic variants associated with Lp(a) lipoprotein level and coronary disease. N Engl J Med.

[35] Chasman DI, Shiffman D, Zee RY (2009). Polymorphism in the apolipoprotein(a) gene, plasma lipoprotein(a), cardiovascular disease, and low-dose aspirin therapy. Atherosclerosis.

[36] Luke MM, Kane JP, Liu DM (2007). A polymorphism in the protease-like domain of apolipoprotein(a) is associated with severe coronary artery disease. Arterioscler Thromb Vasc Biol.

[37] Schunkert H, König IR, Kathiresan S (2011). Large-scale association analysis identifies 13 new susceptibility loci for coronary artery disease. Nat Genet.

[38] Anderson RA, Sando GN (1991). Cloning and expression of cDNA encoding human lysosomal acid lipase/cholesteryl ester hydrolase. Similarities to gastric and lingual lipases. J Biol Chem.

[39] Anderson RA, Rao N, Byrum RS (1993). In situ localization of the genetic locus encoding the lysosomal acid lipase/cholesteryl esterase (LIPA) deficient in Wolman disease to chromosome 10q23.2-q23.3. Genomics.

[40] Zschenker O, Illies T, Ameis D (2006). Overexpression of lysosomal acid lipase and other proteins in atherosclerosis. J Biochem.

[41] Klima H, Ullrich K, Aslanidis C (1993). A splice junction mutation causes deletion of a 72-base exon from the mRNA for lysosomal acid lipase in a patient with cholesteryl ester storage disease. J Clin Invest.

